# Recent advances in microbial production of odd-chain fatty acids

**DOI:** 10.1007/s11274-025-04769-x

**Published:** 2026-01-13

**Authors:** Rudolphus Antonius Timmers, Marta de Vicente, Aurora Rosa-Masegosa, Elvira Romero, Elia Tomás-Pejó, Cristina González-Fernández

**Affiliations:** 1 CARTIF Technology Centre—Area of Circular Economy, Av. Francisco Vallés, 4, 47151 Boecillo Valladolid, Spain; 2 Biotechnological Processes Unit, IMDEA Energy, Madrid, Móstoles 28935 Spain; 3 Faculty of Biological Sciences, Complutense University of Madrid, Madrid, 28040 Spain; 4 Department of Chemical Engineering and Environmental Technology, School of Industrial Engineering, University of Valladolid, Dr. Mergelina, Valladolid, 47011 Spain; 5 Institute of Sustainable Processes, Dr. Mergelina, Valladolid, 47011 Spain

**Keywords:** Odd chain fatty acids, Microbial fermentation, Propionic acid, Pentanoic acid, Heptanoic acid, Pentadecanoic acid and heptadecanoic acid, Genetic engineering

## Abstract

The production of odd-chain fatty acids (OCFAs) is gaining increasing importance due to their diverse applications in food, chemical, and biofuel industries. These fatty acids, which are relatively rare in nature, can be produced from renewable carbon sources through microbial fermentation processes. This review covers the significance of OCFAs in the market and their occurrence, followed by a detailed exploration of their production in mixed and single strain cultures. Specifically, the anaerobic fermentation (AF) conditions and feedstocks used to produce short OCFAs (SOCFAs), such as propionic, valeric, and heptanoic acids are discussed. Additionally, the production of long OCFAs (LOCFAs) by single strains is focusing on yeast, bacteria, and microalgae. Novel approaches for LOCFAs generation from waste carbon sources are also reviewed. This work delves both into the manipulation of microbial communities covering bioaugmentation and process optimization for bioenrichment in open mixed cultures and genetic manipulation in single-strain systems. Finally, the potential for scalable and sustainable production of OCFAs through microbial processes is discussed, as well as the technological advances needed to optimize these pathways.

## Importance in the market and occurrence

Fatty acids are important for the chemical industry because they are versatile, renewable, and highly functional building blocks that can be transformed into a wide range of products. Their long hydrocarbon chains and reactive carboxyl group make them ideal precursors for surfactants, detergents, lubricants, plasticizers, solvents, coatings, and polymers. Fatty acids also allow manufacturers to tune physical properties such as melting point, viscosity, and hydrophobicity by adjusting chain length and saturation. In addition, they can be sourced from renewable biological feedstocks (plant oils, animal fats, microbial fermentation), supporting sustainability and reduced reliance on petrochemicals.

Even-chain fatty acids dominate biological and industrial lipid systems, but odd-chain fatty acids (OCFAs) offer several distinct advantages that are particularly relevant in the context of industrial chemicals. Their terminal propionyl unit gives OCFAs different reactivity, degradation pathways, and metabolic fates compared with even-chain fatty acids, which can be advantageous for designing chemicals. Their unique chain length can alter physical properties such as melting point, crystallinity, and solubility, enabling fine-tuning of lubricants, surfactants, polymers, and coatings. focusing on odd-chain fatty acids helps expand the chemical design space beyond conventional even-chain lipids, supporting innovation in safer, more functional, and more sustainable industrial chemical application.

While even-chain fatty acids dominate in nature, odd-chain fatty acids (OCFAs) are less common and considered to be rarer. OCFAs can be classified based on their chain length in short and long, being short chain those ranging from three to seven carbon atoms and long chain those containing fifteen to nineteen carbon atoms. The production of OCFAs is gaining increasing importance due to their diverse applications in food, chemical, and biofuel industries (Avis et al. 2000; Clausen et al. 2010).

In the case of long OCFAs (LOCFAs), such as pentadecanoic acid (C15:0) and heptadecanoic acid (C17:0), they occur naturally in ruminant fats, dairy products, certain fish, and bacteria that use propionyl-CoA in lipid synthesis. These LOCFAs are gaining attention due to their potential health benefits, including anti-inflammatory and cardioprotective effects. In industry, they are used in the production of biofuels, lubricants, and specialty chemicals, with microbial fermentation emerging as a sustainable production method. Pentadecanoic acid is projected to expand at a compound annual growth rate (CAGR) of 5% (Data Insights Market 2025), while heptadecanoic is registering a 6.5% CAGR from 2026 to 2033 (Verified Market Reports 2025).

Although LOCFAs have greater market value and growing research-driven demand, short OCFAs (SOCFA) are produced in larger total quantities. Among those SOCFAs, propionic acid (HPro) is a versatile building block, mostly used as solvents and flavours (propionate esters), food and feed preservative (calcium propionate), and comonomers for the production of plastics (acrylic fibers). Its global market size ranges 350,000–400,000 metric tons per year, CAGR of 5–6%. Pentanoic acid (HVal, valeric acid) also has a CAGR of about 5–7% but the global market size is lower than that of HPro, ranging 20,000–40,000 metric tons per year. HVal is mainly used as agrochemical, as valerate esters have been shown to be good for crop absorption (Gorbunov et al. 2017; Paul et al. 2025). Similar to propionic, valeric esters (ethyl valerate and butyl valerate) are also used as solvents and plasticizers to improve polymer flexibility. Heptanoic acid (HHep, enanthic acid) exhibits the lowest production, averaging 10,000 metric tons per year, with a modest CAGR of 3–5%. Heptanoic acid is used in the production of esters for perfumes and flavours, as well as in synthetic lubricants, plasticizers, and corrosion inhibitors.

The production of these OCFAs has been most commonly conducted by chemical means. Petrochemical processes to produce SOCFAs include the hydrocarboxylation of ethylene for propionic acid production and the oxidation or hydroformylation of alkanes for HVal and HHep (Gorbunov et al. 2017). Alternatively, a more sustainable production includes the microbial production via fermentation or metabolic engineering. Some bacteria naturally produce SOCFA. This is the case for instance of *Propionibacterium acidipropionici* (renamed as *Acidipropionibacterium acidipropionici*) that produces HPro using glucose or lactate as carbon source through the Wood-Werkman cycle (Chen et al. 2023; Dishisha et al. 2024). In this case, bacteria ferment carbohydrates and subsequently convert succinate to propionate. Another alternative metabolic pathway to produce HPro is the acrylate pathway conducted by some *Clostridium* sp. (Gonzalez-Garcia et al. 2017). In this case, the substrate is lactate, and the intermediate is acrylate formed by the dehydration of lactate. The enzyme acrylate reductase catalyzes, thereafter, the reduction of acrylate, ultimately leading to propionate production. At much lower production yields, *Veillonella parvula* follows the succinate pathway to produce HPro. The reasons for their lower yields are related to their lower levels of energy gained through substrate level phosphorylation and their inability to perform sugars catabolism (Gonzalez-Garcia et al. 2017). To produce pentanoic and heptanoic acids, propionyl-CoA is the primer used by some chain elongating bacteria. *Clostridium kluyveri* and *Megasphaera elsdenii* are the main reported producers by which reverse *β*-oxidation elongates HPro by adding C2 units (acetyl-CoA) into pentanoic and heptanoic acids (Agler et al. 2011; Fernández-Blanco et al. 2023). Likewise, *Megasphaera hexanoica* has also been reported to be able to produce pentanoic and heptanoic acids from a medium with acetate, propionate and fructose (Kim et al. 2019). When dealing with microorganisms that do not produce naturally OCFA, synthetic biology has been also used in model microorganisms (*Escherichia coli*, *Yarrowia lipolytica* or *Clostridium*) (Gonzalez-Garcia et al. 2017; Baur et al. 2022). Some of the approaches to enhance OCFA include the introduction or overexpression of propionyl-CoA generating pathways or the knockout of competing pathways (those favouring the production of acetyl-CoA).

Because the production of OCFAs is gaining increasing importance due to their diverse applications in food, chemical, and biofuel industries, this review provides insights into their natural occurrence, biosynthetic pathways, physiological significance, and emerging strategies for sustainable microbial and biotechnological production.

## Production of SOCFAs in mixed cultures

### Anaerobic fermentation (AF) conditions and feedstocks to produce SOCFAs

The use of anaerobic microbiomes collected from natural environments, wastewater treatments or digesters is emerging as a promising bioprocess for SOCFAs production, as they harness naturally adapted microbial communities. This approach takes advantage of natural syntrophic interactions by which fermentation systems are more efficient in the use of real and complex substrate. Anaerobic microbiomes are composed of bacteria and archaea that breaks down complex organic matter by conducting four-interconnected steps, namely hydrolysis, acidogenesis, acetogenesis and methanogenesis. In the second step, acidogenesis, is where bacteria break the solubilized organic matter into short-chain fatty acids (SCFAs), alcohols, hydrogen and CO_2_, among other intermediates. The diverse microbial community present in open microbiomes enables the use of different feedstocks and provides robustness against operational changes (Greses et al. 2025). Also, relevant is the avoidance of sterile conditions and the use of expensive ways of culturing pure cultures.

When targeting at SOCFAs production, methanogenesis must be inhibited to allow their accumulation rather than being converted into methane. The organic matter is fed into an anaerobic fermenter seeded with an anaerobic microbiome and upon restrictive operation conditions, the effluent produces a rich SOCFAs-stream. To promote the production of SOCFAs, the selection of substrate as well as the operational conditions implemented in the reactors are critical as this would also influence the metabolic pathways that will dominate in the microbial community.

Feeds that promote HPro production include mainly lactate and sugars. Lactate is the carbon source that many propionate producers use via the acrylate pathway (Gonzalez-Garcia et al. 2017). From lactate, the theoretical maximum is 0.67 g propionate/g lactate, assuming a propionate and acetate production in a ~ 2:1 molar ratio (Seeliger et al. 2002). Sugars are also easily converted into pyruvate and then subsequently to succinate. For glucose, an average yield of 0.59 g HPro/g glucose has been reported in real fermentations with single strain cultures (Dishisha et al. 2024). Yet, the amount and nature of this carbon source greatly influence the trade-off between odd and even SCFAs, mostly prevailing the latter (Greses et al. 2022). Because of this, it is important to use complex polysaccharides or real wastes in which a variety of macromolecules are present in the feedstock, whereby a slow glucose release is taking place over fermentation. In fact, the use of amino acid and protein hydrolysates has been also linked to the production of HPro (Regueira et al. 2020; Greses et al. 2022).

With regard to the operational conditions to be implemented in fermenters when targeting at SOCFA, pH in the range of 6–7 (Gonçalves et al. 2024) is preferred over pH of 5.5–6.0 that favour HBu production (Aboudi et al. 2023). In fact, going below pH 4.5 has been shown to be detrimental as succinic acid starts accumulating, indicating an acidogenesis failure (Gonçalves et al. 2024). The simultaneous accumulation of succinic acid and the absence of HPro production in the AF evidences acidogenesis perturbation (Gonçalves et al. 2025a). At pH lower than the p*K*_a_ of the acid, not only a limited microbial community of acid-resistant producers can thrive, but also, they have to face the potential intrusion of the undissociated form of acid that can easily penetrate the cell membrane and decouple internal metabolic reactions. Some other parameters affecting the yields include the organic loading rate, the redox potential and the presence of trace elements. High organic loading rates have been also repeatedly shown to be more effective at accumulating HVal and HPro (Magdalena et al. 2019, 2020; Lago et al. 2025). In the case of redox potential, it has been shown that the lower the redox potential (the more reductive it is), the higher the concentration of OSCFAs. Strategies to control redox potential include gas injection or reagent supplementation. More particularly, threonine and valine are two amino acids that can be used to manipulate redox potential (Regueira et al. 2020). Similar to the addition of amino acids, the presence of cobalt and zinc in the fermentation broth also supported higher SOCFAs production (Dahiya et al. 2020).

The use of mixed wastes is a must when considering the need to treat and upcycle residual streams. As such, the bioconversion efficiency of wastes into SOCFAs is based on the chemical oxygen demand (COD) fed to the anaerobic fermenters and the COD of each SCFA produced. According to the oxidation reaction stoichiometry, COD equivalence for SOCFA is 1.51 for HPro and 2.04 for HVal. Carbohydrate-rich wastes (bioconversion ranging 50% COD-SOCFAs, (Gonçalves et al. 2024; Lago et al. 2025) have been pointed out to support much higher bioconversion into SCFAs than proteins-rich wastes (ranging 30% COD-SCFA (Magdalena et al. 2019). Furthermore, changes in SOCFA profiles distribution are mostly explained in percentage of the total SOCFAs concentration and this might bring misleading conclusions. Because the downstream processing related to the use of SOCFAs is ultimately based on the acid concentration, not only the bioconversion efficiency and distribution profile should be calculated, but the concentration of each acid should be considered. The highest total SOCFAs concentration in anaerobic fermenters operated in continuous feeding mode ranged 25–35 g/L when using carbohydrate-rich wastes (Gonçalves et al. 2025b). Out of this concentration, around 5 g/L can be easily attained for HPro and around 3–5 g/L for HVal. On the other hand, heptanoate is reported in a much lesser extent and concentration of 0.05–0.1 g/L has been recently reported when using real wastes as feedstocks of AF (Reddy et al. 2022; Kurniawan et al. 2024). These concentrations were higher in studies dealing with synthetic media and batch fermentation. For instance, 2 g/L of heptanoate was reported when using fructose as carbon source and both acetate and propionate as electron acceptors in a fermentation inoculated with *Megasphaera* (Jeon et al. 2016). This concentration was even higher (3.6 g/L) when using acetate and pentanoate, probably mediating an easier chain elongation to heptanoate. Although experiments with synthetic media are relevant, as they allow for controlled conditions to isolate specific variables and better understand underlying microbial and biochemical mechanisms, using real wastes in anaerobic fermentation studies is key. Complex composition and variability of real feedstocks, which significantly affect microbial activity and SCFAs production should be the follow up experimentation as this ensures that results are more applicable to real-world conditions.

Alternatively to liquid and solid wastes as feedstocks for SOCFAs, it cannot be neglected that gas streams can also be used. This is the case of syngas that can be employed by some *Clostridium* species to produce HPro via de Wood-Ljungdahl pathway coupled with chain elongation (Devi and Pakshirajan 2025). More specifically, when using this pathway, CO_2_ and carbon monoxide are converted into acetyl-CoA, which is further elongated to propionyl-CoA. Main challenges reported with this approach are the low yield and productivity, as well was the gas mass transfer limitation in fermenters (Ale Enriquez and Ahring 2023).

### Microbial communities for SOCFAs production

Microbial communities present in the AF will depend on the inoculum, substrate composition, reactor configuration, process design, feed system or operational conditions (Xu et al. 2021). For instance, Lv et al. (2022) investigated the effects of pH and hydraulic retention time (HRT) over SOCFAs production using synthetic wastewater with glucose as sole carbon source. Despite the low concentrations of propionate, its production was positively correlated with *Paludibacter*, *Parabacteroides* and a member of *Acidaminococcaceae* family. Huang et al. (2018) used anaerobic digestion sludge as inoculum for investigating the effect of acid and alkali treatment on SOCFAs production. This study reported that HPro and iso-HVal production showed positive correlations with pH 10 and *Desulfovibrio*, *Acinetobacter* and a member of *Candidatus* Aminicenantota phylum and from *Rhodocyclaceae* family. In addition, *Garciella*, *Alkaliphilus*, *Coribacteriaceae* and *Synergistaceae* families and a member of Family XIII AD2011 group were positively related to iso-HVal production. On the other hand, n-HVal production had a positive correlation with extreme pH values (5 and 12) and with some genera such as *Bacteroides*, *Parabacteroides*, *Zoogloea* and *Megasphaera*.

Regarding feedstock, Ma et al. (2017) reported that protein-rich substrate stimulates propionate and valerate production as a consequence of the enrichment of *Clostridia* class. During anaerobic fermentation of *Chlorella vulgaris* biomass (a protein-rich substrate), an increase of HRTs from 4 to 8 h enhanced the population of *Veillonellaceae* family, which is in fact reported as consumer of lactic acid to produce HPro and HVal (Cieciura-Włoch et al. 2020; Llamas et al. 2022). Members of *Ruminococcaceae* and *Moraxellaceae* families have also been reported as producers or HPro from protein-rich substrates (Hao and Wang 2015; Khafipour et al. 2020). In terms of substrates that are difficult to degrade, iso-HVal production was enhanced at pH 9 and 10 under AF of olive mill solid waste, in which *Tissierella*, *Soehngenia*, *Virgibacillus halodenitrificans* and *Clostridiales* order played an important role (Jiménez-Páez et al. 2023). Luo et al. (2021) investigated the application of cellulase to increase AF of paper waste and waste activated sludge. In this case, *Macellibacteroides* and *Bacteroides* genera were identified to be key in propionate production.

In contrast, some microbial groups can also proliferate and consume the targeted SOCFAs. In this sense, syntrophic propionate-oxidizing bacteria, such as *Syntrophobacter*, *Smithella* or *Pelotomaculum*, pose a threat to the accumulation of HPro (Westerholm et al. 2022; Jin and Lu 2023). *Syntrophomonas*, a syntrophic *β*-oxidizer is able to degrade propionate and valerate assisted by hydrogenotrophic methanogens (such as *Methanobacterium*) (Ziemiński and Frąc 2012; Westerholm et al. 2022). Furthermore, *Desulfobulbus* and *Desulfovibrio* are also able to degrade both SCFAs via sulfato-reduction pathway (Westerholm et al. 2022). To prevent the proliferation of these threatening microorganisms, it is recommended to use short HRT, high ammonia concentration, and the control of operational parameters (such as pH or temperature) (Li et al. 2012; Sun et al. 2021; Zhang et al. 2023).

### Process optimization: bioaugmentation

One of the strategies to enhance microbial activity for the accumulation of specific metabolites is bioaugmentation. This methodology refers to the intentional addition of specialized microbial strains or consortia into the indigenous microbial community to enhance process performance towards certain products, stability, or recovery (Atasoy and Cetecioglu 2021a). Bioaugmentation has been mostly used in conventional anaerobic digestion. Yet, a major drawback identified upon this methodology is the struggle to survive or compete of some of the introduced microorganisms with native communities, limiting long-term effectiveness. Additionally, it can add operational complexity and raise regulatory or environmental concerns, particularly when non-native or engineered strains are used. However, developing resilient, well-adapted microbial consortia and optimizing operational conditions can enhance microbial persistence, and provide positive results, as in the examples hereby reported. In the case of improving HPro production, the main microorganism used in bioaugmentation is *A. acidipropionici*. Atasoy and Cetecioglu (2021a) reported an increase of HPro production from about 1 g COD/L in the control to nearly 4 g COD/L with the bioaugmentation strategy, when cheese industry wastewater was used as a substrate. They also noted that bioaugmentation with *A*. *acidopropionici* did not only enhance HPro production, but also total SCFAs production, including iso- and n-HVal. Similarly, another study using the same substrate (Atasoy and Cetecioglu 2021b), but with a bioaugmentation in *Clostridium aceticum* caused a rise in HPro, iso- and n-HVal production. Despite the fact that this microorganism is linked to acetic acid production, the implemented bioaugmentation caused microbial community changes, such as the proliferation of members of *Porphyromonadaceae* family, related to HPro production. Valerate production has been also improved by augmenting the population of *C. kluyveri* in a mixed ruminal inoculum when fermenting switchgrass (Weimer et al. 2015).

## Production of LOCFAs by single strains

LOCFAs are fatty acids that contain an odd number of carbon atoms, typically 15, 17, or 19, rather than the more common even-numbered chains. LOCFAs are closely related to microbial lipids because many microorganisms naturally produce OCFAs during lipid biosynthesis. LOCFAs are usually derived from microbial synthesis, particularly by certain bacteria, yeast and microalgae.

### Yeast

Oleaginous yeasts are capable of accumulating high lipid concentrations (up to 80% w/w), although the fraction represented by LOCFAs still remains lower than 20% w/w (Kolouchová et al. 2015; Tomás-Pejó et al. 2023). LOCFAs accumulation does not only depend on the strain but on the cultivation strategy, substrate composition and metabolic engineering (Park et al. 2018). *Cutaneotrichosporon curvatus*, *Candida* sp., *Rhodotorula toruloides*, and *Y. lipolytica* have been reported as the most promising LOCFAs producers (Kolouchová et al. 2015; Liu et al. 2017; Park et al. 2020; Park and Nicaud 2020b; Qin et al. 2023). Indeed, the versatility of these species to grow both on hydrophobic and hydrophilic substrates widens their utilization for LOCFAs production (Tomás-Pejó et al. 2021). SOCFAs obtained through the AF of organic wastes are a promising carbon source for LOCFAs accumulation (Llamas et al. 2020; de Vicente et al. 2025a). However, the inherent high nitrogen content present in these effluents’ limits lipid accumulation, as low C/N ratios are required to induce lipid synthesis. As such, nitrogen removal from real SCFAs-rich digestates still remains challenging, requiring additional steps, complicating real digestate utilization for LOCFAs at higher operational scales.

LOCFA synthesis relies on the incorporation of propionyl-CoA instead of acetyl-coA via the utilization of 1-propanol, HPro or HVal as a carbon source (Ingram et al. 1977; Park et al. 2018). The inefficient assimilation of these compounds towards lipid synthesis and their inhibitory effects is critical for LOCFA production. High HPro and 1-propanol concentrations may present cell growth inhibition as a result of intracellular acidification, cellular respiration disruption, oxidative stress or changes in membrane permeability, ultimately disrupting the cell growth (Park and Nicaud 2020b; Micalizzi et al. 2021). However, most mechanisms underlying propionate tolerance remain unclear as inhibitory effects vary depending on the strain and operational pH (Gao et al. 2020; Park and Nicaud 2020b). Several *Y. lipolytica*,* R. toruloides*,* Barnettozyma californica*,* Blastobotrys adeninivorans*,* Cutaneotrichosporon oleaginosus* and *Cyberlindnera jadinii* strains can grow at a high HPro concentration of 15 g/L to 30 g/L (Bonzanini et al. 2025; Hermansen et al. 2025), although at a slower growth rate when compared to lower HPro concentrations or higher ones of acetic and butyric acids (Hermansen et al. 2025). Despite the slower growth rates, yeast grown on propionate as sole carbon source can reach similar biomass yield to that obtained with butyric acid or even up to 45% higher biomass yield than when using acetic acid (Hermansen et al. 2025). Tabaa Chalabi et al. (2024) reported a LOCFAs increment from 8.7% in the absence of sodium propionate to 68% when using 5 g/L HPro as carbon source. However, higher concentrations inhibited yeast growth. To avoid HPro inhibitory effects, both fed-batch strategies and supplementation via pulse additions have been evaluated. In the same sense, co-feeding HPro with an easily assimilable carbon source such as glucose or acetic acid reported an increased growth and higher total lipid yields (with higher OCFAs concentrations) (Kolouchová et al. 2015; Park et al. 2021; de Vicente et al. 2025a). In this regard, the importance of finding a balance between acetyl-CoA and propionyl-CoA has been highlighted to be key when targeting LOCFAs accumulation (de Vicente et al. 2025a).

The highest percentages of LCFAs reported in literature have been achieved using *R. toruloides* and *B. californica*, reaching nearly 90% of the total fatty acids when grown on 15 g/L HPro. However, overall lipid yield remained moderate. For example, *C. oleaginosus* achieved a LOCFAs yield with regard to the consumed HPro of 0.07 g/g (Bonzanini et al. 2025).The rest of the strains tested in the same study (*C. oleaginosus*,* Y. lipolytica*, and *B. californica*) accumulated between 35 and 80% LOCFAs, but with LOCFAs yields lower than 0.02 g/g. *Y. lipolytica* has reached a maximum accumulation of up to 67% LOCFAs for genetically engineered strains (Park et al. 2021; de Vicente et al. 2025a). Other species, C. *curvatus*,* Candida* sp. and *C. jadinii* have shown to be able to accumulate 67.7, 37 and 37% OCFAs, respectively, under different SCFAs concentrations (Kolouchová et al. 2015; Krikigianni et al. 2022; Hermansen et al. 2025).

### Bacteria

Bacteria do not present a multi-enzymatic complex type I fatty acid synthase (FAS) like yeast, but a type II system with independent enzymes (Schweizer and Hofmann 2004; Runguphan and Keasling 2014). For example, the enzyme *β*-ketoacyl-ACP synthase III (KAS III, *fab*H) present in type II FAS, is involved in the initial step of FA biosynthesis and chain elongation, by incorporating propionyl-CoA instead of acetyl-CoA (Zhang et al. 2020; Qin et al. 2023). As in yeast, substrate flexibility set the basis for exploring the utilization of HPro for LOCFAs synthesis (Wu and San 2014a, b; Zhang et al. 2020). Besides these differences, yeast and bacteria present homologous OCFAs accumulation pathways. Bacteria are also capable of accumulating high LOCFAs concentrations. However, their potential depends on the utilization of different carbon sources and the presence of different enzymes and mechanisms for lipid biosynthesis. Moreover, bacteria could be easily engineered with the stablished molecular tools, raising the interest in their utilization (Wu and San 2014b). Most bacteria able to accumulate LOCFAs belong to the Actinomycetota phylum (e.g., *Rhodococcus* sp.) and *E. coli* (model organism for engineering) (Janßen et al. 2013; Bhatia et al. 2019).

In the presence of HPro up to 62% w/w LOCFAs were accumulated in *Rhodococcu*s sp. YHY01 (Bhatia et al. 2019). However, since nitrogen plays a critical role in fatty acids accumulation, medium optimization and co-fermentation approaches are needed. Bhatia et al. (2019) revealed that a glycerol, propionate and ammonium chloride mixture (0.32%:0.76%:0.040% w/v) could increment fatty acids accumulation up to 69% w/w (1.3 g/L), with a content of 85% LOCFAs.

Propanol-1 can also be used as carbon source for LOCFAs production. *Rhodococcus opacus* in minimal salt medium under a mixture of 12 g/L glucose and 0.5–1.5% v/v 1-propanol, achieved 1.31–1.61 g/L of total fatty acids, with an LOCFAs content of 76.2% and 84.5% (Zhang et al. 2019). The maximum LOCFAs concentration in bacteria was achieved by the same strain after enhancing the precursors pool, achieving 68.2% (1.87 g/L) LOCFAs by co-feeding 24 g/L of glucose and 6.5 g/L 1-propanol (Chu et al. 2020).

*E. coli* does not naturally accumulate high lipid concentration, however metabolic engineered strains (e.g., HWK201) have allowed to reach up to 1.2 g/L with 85% LOCFAs in the form of C15 (Wu and San 2014a). As in yeast and despite the high OCFAs concentrations, LOCFAs yields in bacteria in terms of g/L remain much lower than those of total fatty acids. Engineered *E. coli and Rhodococcus* strains can accumulate up to 30 g/L and 19.5 g/L of total fatty acids, whereas the maximum LOCFAs accumulation remains lower than 2–3 g/L under nitrogen limitation (Voss and Steinbüchel 2001; Fang et al. 2021). Therefore, further optimization of culture media, fermentation strategies and metabolic engineering approaches are still needed.

### Microalgae

The accumulation pathways in the case of microalgae remain poorly explored as LOCFAs are more rarely accumulated than in yeast or bacteria. In microalgae, LOCFAs have been used as markers of bacterial contamination (Grubišić et al. 2022). The low LOCFAs concentrations in microalgae are due to the utilization of CO_2_ as main carbon source. CO_2_ enters the Calvin cycle and gets converted into acetyl-CoA, that sequentially enters the FAS, mainly leading to the accumulation of even chain fatty acids (Zeng et al. 2011). OCFAs production follows the same FAS pathway as in yeast (type I), however the metabolic pathways and enzymes involved in algae are less well characterized.

Fatty acids production in microalgae has been focused on docosahexaenoic acid (DHA, C22:6n3) (Ma et al. 2023). Therefore, general efforts have been directed at enhancing fatty acids metabolic precursors such as acetyl-CoA and malonyl-CoA (Xue et al. 2021). Although this strategy boosts total fatty acids content, it does not translate into higher LOCFAs yield. The maximum LOCFAs content reported from CO_2_ in synthetic medium was 22% of C17:0 in *Picochlorum* sp., within 8% w/w lipids (Grubišić et al. 2022).

In microalgae, high concentrations of propionyl-CoA precursors also have inhibitory effects on cell growth. Therefore, as in yeasts and bacteria, the supply of propionyl-CoA and its consumption pathways must be balanced to selectively enhance LOCFAs accumulation.

Heterotrophic and mixotrophic strains have arisen as promising alternatives for LOCFAs production since they can utilize reduced organic substrates, rather than exclusively CO_2_ (Oliver et al. 2022). In this sense, several studies have evaluated microalgae growth on different SCFAs and specific amino acids (valine, methionine, threonine and isoleucine). Out of these compounds, the use of propionate as carbon source resulted in LOCFAs increase of 3.5-fold in *Schizochytrium* sp. When grown on real waste-derived digestates rich in SCFAs, *Schizochytrium limacinum* accumulated 25% LOCFAs out of the total lipids (54% w/w), accounting for 2.5 g/L OCFAs (Oliver et al. 2022). As also seen for yeasts and bacteria, microalgae show a prevalence for acetic and butyric acids in terms of carbon sources for LOCFAs production, with a faster consumption rate of those SOCFAs when compared to HPro (Oliver et al. 2022).

### Novel approaches for LOCFAs from residual carbon sources

Thanks to recent advances in synthetic biology, efficient LOCFA production has made significant progress by optimizing or assembling microbial biosynthetic pathways. Most of these strategies enhanced propionyl-CoA pools, which can replace acetyl-CoA in the first condensation step of fatty acids synthesis. Many studies have also proved the efficacy of combining metabolic, media composition and bioprocess engineering. Regarding the carbon source, glucose and propionate have been often used but there are also a few examples using low-cost industrial by-products such as glycerol and sugar beet molasses. In cases using SOCFAs as carbons sources, most studies have used commercial propionate but there is also a seminal work using real digestate from AF (de Vicente et al. 2025a).

**Fig. 1:**
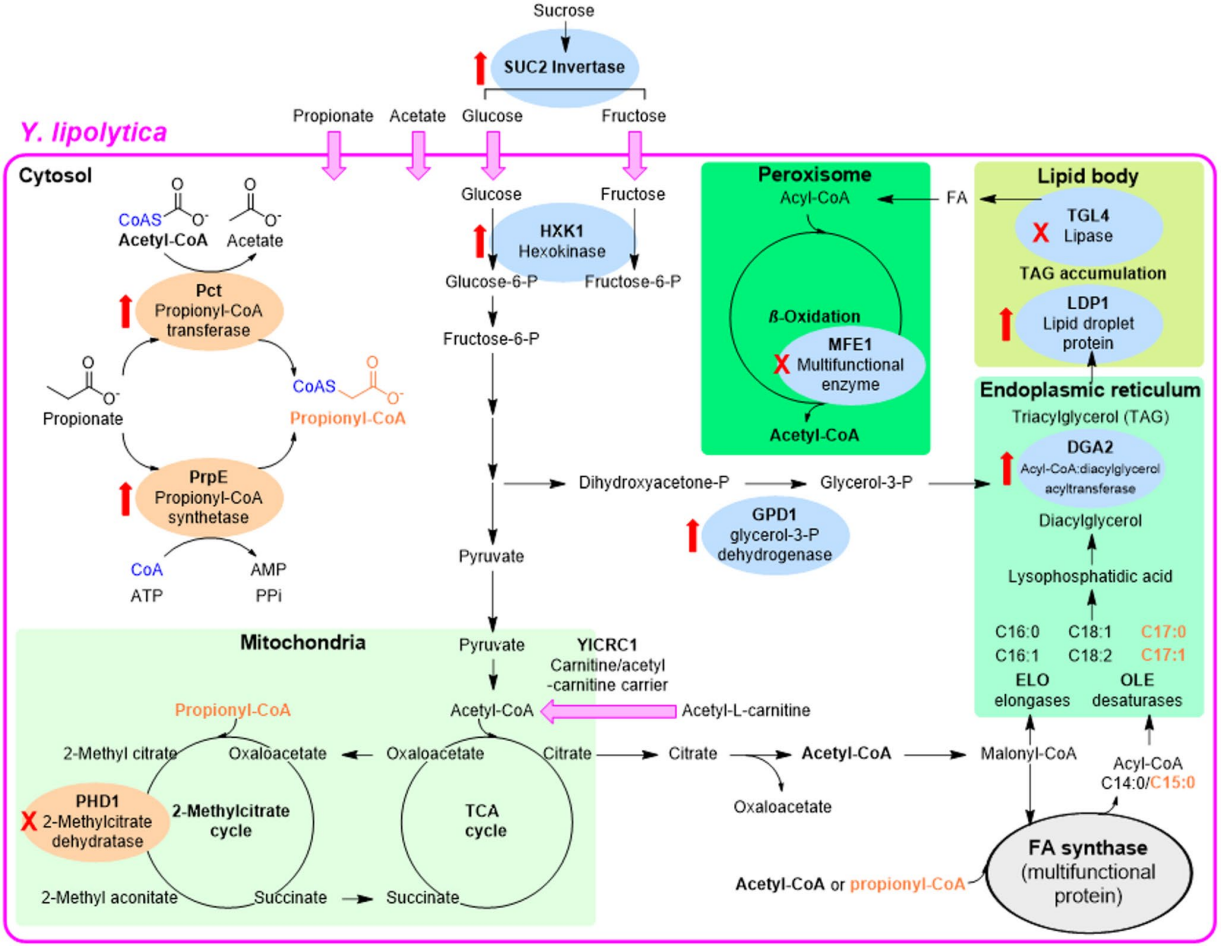
Engineered metabolic pathways in *Y. lipolytica* for synthesis of OCFA in obese strains. Enzymes overexpressed or knocked out are indicated with a red arrow or cross, respectively. Engineered enzymes for propionyl-CoA accumulation are shown in orange ellipses, while those for enhancing total lipids (obese strains) are in blue ellipses

Most recent engineering studies for LOCFAs production have exploited yeasts or demonstrated the great potential of microalgae. However, pioneering studies were performed using engineered *E. coli*. This host was used to overexpress *Salmonella enterica* propionyl-CoA synthetase (*Se*prpE) and *Umbellularia californica* acyl-ACP thioesterase (*Uc*TE) producing 0.276 g/L LOCFAs with a ratio of 23.4% of the total free fatty acids using glucose and propionate (Wu and San 2014b). *Se*prpE converted propionate into propionyl-CoA, while *Uc*TE terminated fatty acyl group extension (Wu and San 2014b). More recently, almost 47% LOCFAs proportion (0.17 g/L LOCFAs) was achieved for *Y. lipolytica* using glucose and propionate by knocking out PHD1 gene in 2-methyl citrate pathway, which prevented propionyl-CoA processing and thereby, leading to an increased propionyl-CoA pool for LOCFAs synthesis (Fig. 1) (Park et al. 2018). After that, various mutations were added, resulting in a new obese strain with increased total lipid content accumulation (Table 1)(Park et al. 2018). A further engineered obese strain, Δphd1 obese-L (Park and Nicaud 2020a) (Table 2; **41**.9% LOCFAs proportion) was used in a subsequent study to investigate various propionate-activating genes. Best results (> 54% LOCFAs proportion) were obtained for obese-L strains overexpressing: (i) Cppct or Repct (propionyl-CoA transferase from *Clostridium propionicum* or *Ralstonia eutropha*, respectively), which transfer CoA generally from acetyl-CoA to propionate; or (ii) *Se*prpE (propionyl-CoA synthetase described above). Among them, Repct obese-L was the top-performing strain having a 61.7% LOCFAs proportion using glucose, propionate and acetate. In addition, this study showed the importance of optimizing the propionate: acetate ratio to simultaneously maximize the LOCFAs proportion, total lipid content, and cells growth, having best result with 0.5% propionate, 1% acetate and 2% glucose (Park et al. 2021).

**Table 1:** *Y. lipolytica* strains used in studies for enhancing LOCFAs production

Strain	Description	Abbreviation	Ref.
JMY3776	Δphd1 Δmfe1 Δtgl4 pTEF-DGA2-LEU2ex pTEF-GPD1-URA3ex	Δphd1 obese	(Park et al. 2018)
JMY3820	Δpox1-6 Δtgl4 pTEF-DGA2 pTEF-GPD1	Δpox1-6 obese	(Lazar et al. 2014)
JMY7228	Δphd1 Δmfe1 Δtgl4 pTEF-DGA2 pTEF-GPD1 hp4d-LDP1-URA3ex	Δphd1 obese-L	(Park and Nicaud 2020a)
JMY7412	MY3820 GGV-AAT2 GGV-THR1-THR4-ILV1 GGV-HOM3-HOM2-HOM6-URA3ex LEU2	Obese-ATH	(Park et al. 2020)
JMY7780	JMY7228 pTEF-Repct-LEU2ex	*Re*pct obese-LP	(Park et al. 2021)
JMY7782	JMY7228 pTEF-SeprpE-LEU2ex	*Se*prpE obese-LP	(Park et al. 2021)
JMY7877	JMY7780 pTEF-ScSUC2-LEU2ex pTEF-YlHXK1-URA3ex	*Re*pct obese-LPSH	(Al Sahyouni et al. 2022)
JMY8438	JMY7228 pTEF-Repct-LEU2ex pTEF-RebktB-Hygroex	Obese-LPB	(Park et al. 2021)
JMY9178	JMY7877 pTEF-YlDGA2-URA3ex pTEF-YlOLE1-LEU2ex	YlDGA2 YlOLE1 obese-LPSH	(Tabaa Chalabi et al. 2024)

**Table 2:** Examples of metabolic engineering to enhance LOCFAs in various microbial hosts

	Species	Carbon source, scale, conditions	Strategy	LOCFAs proportion (%), main LOCFA (CX: X), titer LOCFA (g/L)	Ref.
BACTERIA	*E. coli*	Glucose, propionate, flasks, 30 °C, 250 rpm	Overexpression *Se*prpE, *Uc*TE	23, C13:0, 0.3	(Wu and San 2014b)
YEASTS	*Y. lipolytica*	Glucose, 50 mL, 250 mL flasks, 28 °C, 180 rpm	Obese-ATH, propionyl-CoA via threonine, overexpression 7 genes	6, C17:1, 0.4	(Park et al. 2020)
		Glucose, propionate, 50 mL, 250 mL flasks, 28 °C, 180 rpm	∆PHD1 obese	42, C17:1, 0.6	(Park et al. 2018)
		Glucose, propionate, acetate, 50 mL, 250 mL flasks, 28 °C, 180 rpm	*Re*pct *Re*bktB obese-L	62, C17:1, 1.9	(Park et al. 2021)
		Glucose, real SCFA-rich digestate, 100 mL, 250 mL flasks, 28 °C, 180 rpm	*Se*prpE obese-L	51, C17:1, 2.0	(de Vicente et al. 2025a)
		Sugar beet molasses, glycerol, acetate, propionate, 100 mL, 500 mL flasks, 28 °C, 170 rpm	*Yl*DGA2 *Yl*OLE1 *Re*pct obese-LPSH	67, C17:1, N/A	(Tabaa Chalabi et al. 2024)
	*S. cerevisiae*	Glucose, 10 mL, 100 mL flasks, 30 °C, 250 rpm	Propionyl-CoA via threonine, overexpression 6 genes, non-native fatty acid synthase	52, C17:1, 0.3	(Meng et al. 2025)
		Glucose, 100 mL, 500 mL flasks, 30 °C, 120 rpm	Propionyl-CoA via alanine, overexpression 8 genes	20, C17:1, 0.1	(Qi et al. 2023)
		Glucose, 10 mL, 100 mL flasks, 30 °C, 220 rpm	Overexpression oleate 12-hydroxylase or a hydratase, alcohol dehydrogenase, Baeyer-Villiger monooxygenase, lipase	N/A, N/A, 0.04 (C7:0 + *ω*-OH-C11 + C9:0 + *ω*-OH-C9)	(Dong et al. 2024)
MICROALGAE	*Schizochytrium* sp. 31	Glucose, flasks, 25 °C, 180 rpm	Overexpression malic enzyme, *ELO3*	N/A, C15:0 + C17:0, 3.3	(Wang et al. 2019)
	*S. limacinum* SR21	Glucose, 50 mL, 250 mL flasks, 28 °C, 200 rpm	Overexpression FAS and PKS pathways	19, C15:0, N/A	(Duan et al. 2025)
	*Schizochytrium* sp. HX-308	Glucose, propionate, corn powder, 100 mL, 500 mL flasks, 28 °C, 170 rpm	Deletion methylmalonyl-CoA mutase	20, C16:0, 2.8	(Ma et al. 2023)

More recently, *Re*pct obese-LP and *Se*prpE obese-LP have been implemented for LOCFAs production using real AF digestates (C2-C6 SOCFAs) (de Vicente et al. 2025a). *Se*prpE obese-L strain produced the highest LOCFAs proportion (50.7%), similarly to the results with synthetic media (de Vicente et al. 2025a). Thus, by using real digestates, this work has facilitated the development of more cost-effective, sustainable and scalable bioprocesses. Furthermore, LOCFAs proportion reached 60.4% by adding an additional acetate pulse after its exhaustion, promoting a more rapid depletion of residual SOCFAs from the digestate (de Vicente et al. 2025a).

*Re*pct obese-LP was further engineered for sucrose assimilation by overexpressing *S. cerevisiae* invertase SUC2 and *Y. lipolytica* hexokinase HXK1 (Al Sahyouni et al. 2022). The resulting strain, *Re*pct obese-LPSH, yielded 58% LOCFAs proportion in fermentations with sugar beet molasses, glycerol and propionate (El Kantar and Koubaa 2022). More recently, *Re*pct obese-LPSH was engineered to overexpress Δ9 fatty acid desaturase (*Yl*OLE1, introduces double bond in FA) and diacylglycerol O-acyltransferase 2 (*Yl*DGA2, incorporates third acyl-CoA onto diacylglycerol) achieving 67% LOCFA proportion using sugar beet molasses, glycerol, acetate and propionate (Tabaa Chalabi et al. 2024). Membrane transport engineering was also studied by modifying the expression level of *Y. lipolytica* mitochondrial carnitine/acetyl-carnitine carrier (YlCRC1) that transports peroxisomal or cytosolic acetyl-CoA to mitochondria to enter TCA cycle (Messina et al. 2023). Using glucose, propionate and acetate, the strain overexpressing *Re*pct presented around a 3-fold decrease in LOCFAs proportion when YlCRC1 gene was deleted in its genome (Messina et al. 2023). This result may due to a higher concentration of cytosolic acetyl-CoA, which competes with propionyl-CoA in first condensation step of FAs synthesis (Messina et al. 2023).

Recently, increased biosynthesis of LOCFA from glucose without propionate supplementation has been shown for the first time in *Y. lipolytica* by up-regulating the threonine biosynthesis (Park et al. 2020) as previously shown in *E. coli* (Tseng and Prather 2012). This strategy required the overexpression of seven *Y. lipolytica* genes involved in the native metabolic pathway by which oxaloacetate (from glucose) was converted into *α*-ketobutyrate via threonine. Then, *α*-ketobutyrate was converted to propionyl-CoA by the native pyruvate dehydrogenase (PDH) complex. The engineered strain (ATH) and the obese counterpart (obese-ATH) produced 3.9% and 5.6% LOCFA proportion, respectively, with regard to 0.8% attained by the wild type (Table 2).

Even though *Y. lipolytica* is the yeast most investigated, there are also a few examples of LOCFAs production using engineered *S. cerevisiae*. For example, a high OCFAs proportion (51.9%) was achieved by overexpressing three genes in native threonine biosynthetic pathway from glucose and two foreign genes coding for enzymes to convert this amino acid into propionyl-CoA (Meng et al. 2025). In addition, this strain overexpressed a non-native fatty acid synthase having higher affinity for propionyl-CoA than *S. cerevisiae* counterpart (Meng et al. 2025). In another study with *S. cerevisiae*, a 20-fold increase in LOCFAs proportion was achieved by combining and optimizing two previously engineered pathways with eight overexpressed non-native enzymes (Qi et al. 2023). Specifically, the first pathway produced 3-hydroxypropionic acid from glucose via *β*-alanine(Borodina et al. 2015), while the second one converted 3-hydroxypropionic acid into propionyl-CoA (Krink-Koutsoubelis et al. 2018). In another example, *S. cerevisiae* was engineered to produce medium-length OCFAs from glucose (Dong et al. 2024). The pathway involved enzyme-based hydroxylation of fatty acids at a specific position that stablishes the length of the resulting LOCFA. Next, overexpressed alcohol dehydrogenase formed the corresponding keto fatty acids, which served as substrates for a recombinant Baeyer-Villiger monooxygenase producing the esters to be hydroxylated by overexpressed lipase. This yielded 8–13 mg/L of heptanoic, 11-hydroxyundec-9-enoic, nonanoic, and 9-hydroxynonanoic acids.

Regarding engineered microalgae for LOCFAs production, *Schizochytrium* strains (a fungus-like heterotrophic unicellular marine eukaryote) has gained great attention due to its remarkable lipid synthesis. In *Schizochytrium limacinum* SR21, almost 2-fold increase in LOCFAs proportion (reaching 13.77 and 5.46% for pentadecanoic acid and heptadecanoic acid, respectively) was attained by engineering the FA synthase and polyketide synthase pathways (Duan et al. 2025). Similarly, a previous study on *Schizochytrium* sp. S31 yielded a strain with increased accumulation of both polyunsaturated fatty acids and LOCFAs proportion, as a result of enhancing NADPH, acetyl-CoA and propionyl-CoA pools, owing to the heterologous overexpression of the NADPH-producing malic enzyme and an elongase to convert C16 FA into C18 FA preventing acetyl-CoA carboxylase inhibition by C16 FA (Wang et al. 2019). In *Schizochytrium* sp. HX-308, knocking out the methylmalonyl-CoA mutase (MCM) gene resulted in a 6-fold increase in LOCFAs proportion and titter (20.2% and 2.82 g/L, respectively) (Ma et al. 2023). The MCM is a key enzyme in the native methylmalonyl pathway that causes the propionyl-CoA consumption in the TCA cycle instead of the FAS pathway for LOCFA accumulation (Ma et al. 2023). The titter was further increased to 6.8 g/L by a fed-batch co-feeding strategy preventing propionate toxicity, while isoleucine or valine promoted LOCFAs biosynthesis via propionyl-CoA formation. Thus, this work showed the benefits of combining metabolic, medium composition and bioprocess engineering.

## Outlook and future perspectives

The microbial production of OCFAs has emerged as a highly promising biosynthesis technology, offering applications in pharmaceuticals, nutraceuticals, biofuels, and specialty chemicals. Because even-chain fatty acids are more often found in nature and reported in scientific literature, a specific effort should be directed towards the research devoted to the production of OCFAs. Contrary to even-chain fatty acids, the unique chemical structure of OCFAs confers them specialized functional and physicochemical properties that make them particularly valuable for the synthesis of high-value products such as flavour compounds, lubricants, and precursors for polymer synthesis. Recent advances in metabolic engineering, synthetic biology, and bioprocess optimization have enabled significant improvements in OCFA yield and productivity in microorganisms’ species, including *E. coli*, *Y. lipolytica*, *S. cerevisiae* or anaerobic microbial consortia. These developments underscore the potential of microbial cell factories as versatile and sustainable platforms for tailored OCFAs production.

Despite these advances and the increased research efforts in the last years, the field still faces several challenges. Key bottlenecks include the limited availability of precursors such as propionyl-CoA, methylmalonyl-CoA, and other odd-chain building blocks. Redox imbalance, by-product formation, and regulation of competing pathways often reduce process efficiency. Besides, scalability and economic feasibility of OCFAs microbial-based production processes remain critical barriers. Achieving industrial-scale production requires not only robust and high-yielding strains or consortia but also low-cost and sustainable feedstocks, optimized cultivation conditions, and efficient downstream processing for extraction and purification.

To make OCFA available as a chemical platform, an adequate concentration, extraction and purification is required (Tomás-Pejó et al. 2023). SCFAs produced in AF require a multi-step recovery. First, biomass is removed via centrifugation and microfiltration followed by flow-electrode capacitive deionization, electrodialysis, ion exchange, adsorption or membrane contactors, which allow SCFAs separation from ammonium, phosphate and other nutrients present in the digestate (Sun et al. 2023; Chen et al., 2025, Polat et al. 2025). In occasions, solvent-based extraction is carried out to concentrate the SCFAs for sequential recovery steps (Oh et al. 2022; Polat et al. 2025).

By contrast, LOCFAs are primarily retained within the microbial cells rather than secreted into the culture broth requiring a cell disruption step. Similar challenges arise in this context, as cell disruption, extraction, and recovery face comparable limitations (de Vicente et al. 2025b). Disruption methods can be classified in mechanical (e.g. sonication, high pressure homogenization (HPH), bead milling) and non-mechanical (physical, chemical or enzymatic). The selection of the appropriated method depends on the microorganism, product of interest and industrial scale. Among these, HPH, sonication and acid hydrolysis are shown to be the most effective disruption methods (de Vicente et al. 2025b). Cell disruption is followed by solvent extraction, conventionally chloroform/methanol, although the use of green solvents (e.g. alcohols, esters, ethers and switchable solvents) has gained recent attention (de Vicente et al. 2025b). Other approaches include calcium-based precipitation, direct solvent-based extraction or the use of membranes with molecular selectivity (Pervez et al. 2022). Extracted of concentrated long and SCFAs can be purified by distillation as their boiling point increase with the carbon chain length. Distillation is recommended for low SCFAs concentrations as high concentrations may exhibit azeotropic behaviour that limits efficient separation (Pervez et al. 2022). However, the separation between even and OCFAs remains challenging due to their similar properties (Jänisch et al. 2019). In this sense, even and OCFAs separation mainly relies on urea complexation or fractional crystallization followed by purification using vacuum distillation thanks to their varying melting points and solubility properties (Cermak et al. 2012; Gonzalez-Fernandez., 2020). Most existing technologies for these purposes are reported to be costly or energy-intensive, making them less competitive with conventional petrochemical production routes.

**Fig. 2:**
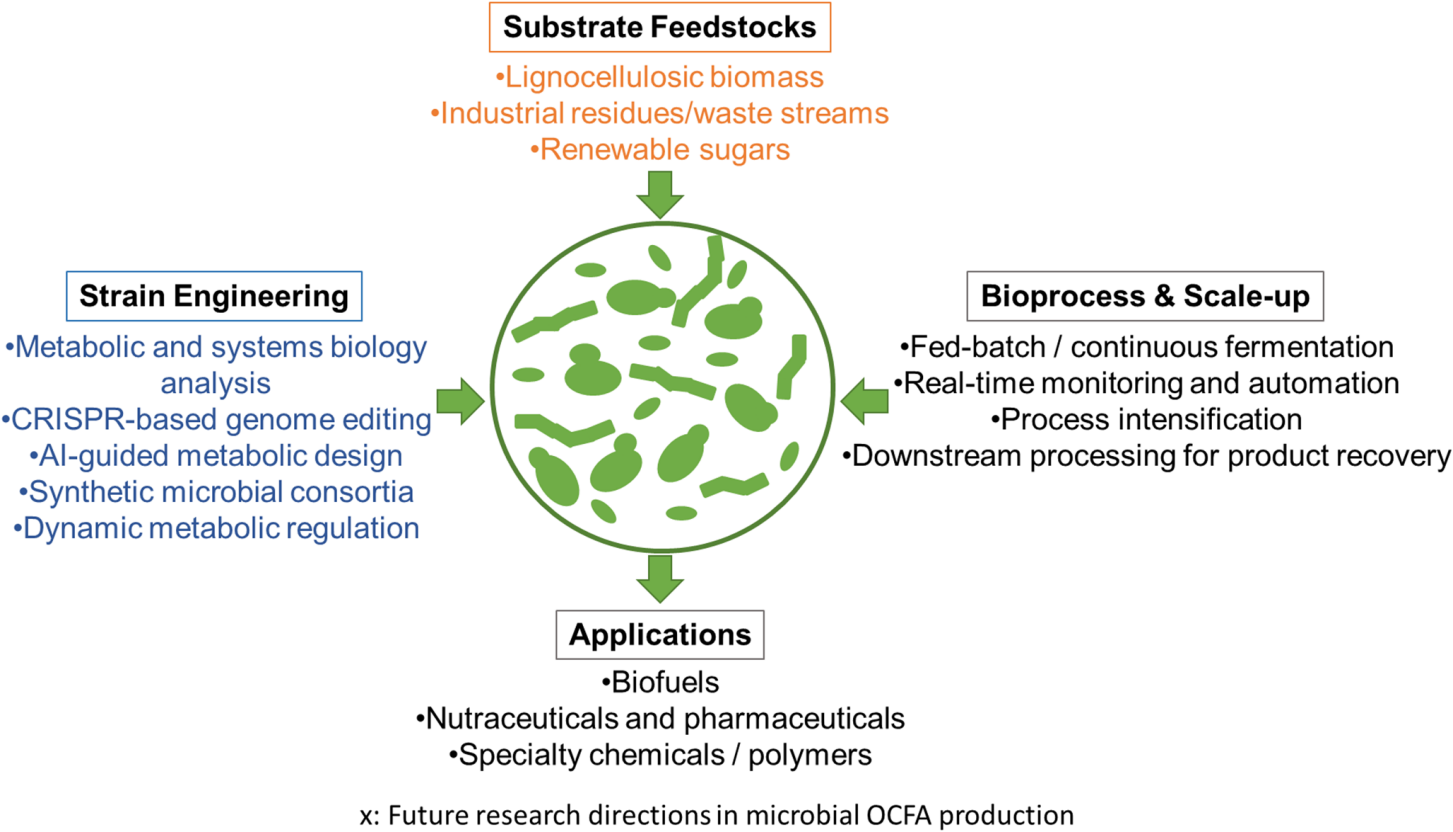
Alternative directions to continue with microbial OCFAs production

Nevertheless, in the current context of climate change, evaluating bioprocesses solely on economic grounds overlooks their potential to substantially reduce greenhouse gas emissions and mitigate overall environmental impact. Addressing these challenges demands an integrated approach that combines advanced genetic engineering, systems biology, and innovative bioprocess design and engineering. Several research directions are particularly promising to move forward an efficient microbial production of OCFAs (Fig. 2).

First, comprehensive metabolic and systems biology analyses are essential to unravel the complex regulatory networks and flux distributions governing OCFA biosynthesis. Genome-scale metabolic models can predict bottlenecks, guide rational strain design, and identify strategies to redirect carbon flux toward OCFA synthesis. Second, CRISPR-based genome editing and AI/ML-driven metabolic engineering provide unprecedented opportunities for precision single strain optimization. These tools allow targeted modifications of key enzymes, regulators, and transporters, enabling higher precursor availability, balanced cofactor regeneration, and improved OCFA yields. Third, the exploration of alternative carbon sources, including lignocellulosic biomass, industrial residues, and waste streams, can enhance the sustainability and economic competitiveness of microbial OCFA production. Utilizing these renewable substrates not only reduces reliance on conventional carbon sources (sugars) but also contributes to circular bioeconomy approaches.

Exploiting well-established microbial consortia allows the distribution of distinct metabolic tasks across different microorganisms, which can enhance substrate utilization, alleviate the metabolic burden on individual strains, and improve overall process stability and robustness. For example, one microorganism could convert complex substrates into short-chain precursors, while another specializes in elongation and OCFA synthesis. Thus, another promising avenue is the development of synthetic microbial consortia or co-cultivation systems. Dynamic metabolic regulation strategies, such as inducible promoters, biosensors, and feedback control, can further optimize production in response to changing environmental or process conditions. Neural networks and microbial population dynamics can shed light on the microbial interaction leading to increase product yields particularly in the case of microbial consortia for short OCFAs. Integrating these cutting-edge technologies will provide a roadmap to overcome current limitations and unlock the full potential of microbial OCFA production.

While several single strains have shown high proportions of LOCFAs, mixed-culture anaerobic fermentation of waste streams currently offers a promising pathway toward achieving economically relevant volumetric productivity at industrial scale due to its potential to operate at relatively high substrate loading rates, under robust and non-sterile conditions. In contrast, pure-culture platforms, although capable of achieving higher LOCFA selectivity under controlled conditions, may be subject to limitations related to product inhibition, sterility requirements, and scale-up complexity, which can affect their volumetric productivity at industrially relevant scales. Mixed-culture systems may be more readily integrated into existing anaerobic digestion infrastructure, potentially enabling process intensification and improved techno-economic performance.

On the bioprocess front, continuous fermentation systems, real-time monitoring, and process automation offer opportunities for efficient, scalable, and reproducible OCFA production. Process intensification strategies, including fed-batch and perfusion systems, can maintain optimal nutrient levels, control byproduct accumulation, and enhance volumetric productivity. Coupling microbial OCFA production with downstream conversion into value-added products (e.g., specialty chemicals, or functional lipids) can create integrated biorefinery platforms, increasing overall economic viability.

Nevertheless, it should be noted that further pilot-scale validation and comprehensive techno-economic analyses are required to fully assess and compare the industrial feasibility of the different production platforms.

## Conclusion

Microbial OCFA production remains in its early stages compared to even-chain fatty acids, but the combination of modern tools, innovative strategies, and interdisciplinary research makes the field highly promising. By addressing key challenges in strain engineering, substrate utilization, metabolic regulation, and process design, microbial platforms could deliver sustainable, economically feasible, and versatile production of OCFAs for diverse industrial applications. The growing regulatory pressure on petrochemicals opens the market to bio-based alternatives. However, continued investment in research and development will be essential to establish OCFAs as major bio-based platform molecules, contributing to both scientific advancement and a more sustainable bioeconomy.

## Data Availability

No datasets were generated or analysed during the current study.

## References

[CR1] Aboudi K, Greses S, González-Fernández C (2023) Hydraulic retention time as an operational tool for the production of Short-Chain carboxylates via anaerobic fermentation of Carbohydrate-Rich waste. Molecules 28:6635. 10.3390/molecules28186635 37764411 10.3390/molecules28186635 PMC10537262

[CR2] Agler MT, Wrenn BA, Zinder SH, Angenent LT (2011) Waste to bioproduct conversion with undefined mixed cultures: the carboxylate platform. Trends Biotechnol 29:70–78. 10.1016/j.tibtech.2010.11.006 21190748 10.1016/j.tibtech.2010.11.006

[CR3] Al Sahyouni W, El Kantar S, Khelfa A et al (2022) Optimization of cis-9-heptadecenoic acid production from the oleaginous yeast. Yarrowia Lipolytica Ferment 8:245. 10.3390/fermentation8060245

[CR4] Ale Enriquez F, Ahring BK (2023) Strategies to overcome mass transfer limitations of hydrogen during anaerobic gaseous fermentations: A comprehensive review. Bioresour Technol 377:128948. 10.1016/j.biortech.2023.128948 36963702 10.1016/j.biortech.2023.128948

[CR5] Atasoy M, Cetecioglu Z (2021a) Bioaugmentation as a strategy for tailor-made volatile fatty acid production. J Environ Manage 295:113093. 10.1016/j.jenvman.2021.113093 34167052 10.1016/j.jenvman.2021.113093

[CR6] Atasoy M, Cetecioglu Z (2021b) Bioaugmented mixed culture by *Clostridium aceticum* to manipulate volatile fatty acids composition from the fermentation of cheese production wastewater. Front Microbiol 12. 10.3389/fmicb.2021.658494 10.3389/fmicb.2021.658494 PMC8446653 34539589

[CR7] Avis TJ, Boulanger RR, Bélanger RR (2000) Synthesis and biological characterization of (Z)-9-heptadecenoic and (Z)-6-methyl-9-heptadecenoic acids: fatty acids with antibiotic activity produced by *Pseudozyma flocculosa*. J Chem Ecol 26:987–1000. 10.1023/A:1005464326573

[CR8] Baur T, Wentzel A, Dürre P (2022) Production of propionate using metabolically engineered strains of *Clostridium Saccharoperbutylacetonicum*. Appl Microbiol Biotechnol 106:7547–7562. 10.1007/s00253-022-12210-8 36282302 10.1007/s00253-022-12210-8 PMC9666320

[CR9] Bhatia SK, Gurav R, Choi T-R et al (2019) A clean and green approach for odd chain fatty acids production in *Rhodococcus* sp. YHY01 by medium engineering. Bioresour Technol 286:121383. 10.1016/j.biortech.2019.121383 31071574 10.1016/j.biortech.2019.121383

[CR10] Bonzanini V, Haddad Momeni M, Olofsson K et al (2025) Impact of glucose and propionic acid on even and odd chain fatty acid profiles of oleaginous yeasts. BMC Microbiol 25:79. 10.1186/s12866-025-03788-w 39966733 10.1186/s12866-025-03788-w PMC11834278

[CR11] Borodina I, Kildegaard KR, Jensen NB et al (2015) Establishing a synthetic pathway for high-level production of 3-hydroxypropionic acid in *Saccharomyces cerevisia*e via β-alanine. Metab Eng 27:57–64. 10.1016/j.ymben.2014.10.003 25447643 10.1016/j.ymben.2014.10.003

[CR12] Cermak SC, Evangelista RL, Kenar JA (2012) Distillation of Natural Fatty Acids. *Distillation: Advances from Modeling to Applications*, 109

[CR13] Chen Z, Zhu S, Gao S et al (2023) A hyperthermophilic anaerobic fermentation platform for highly efficient short chain fatty acids production from thermal hydrolyzed sludge. Water Res 243:120434. 10.1016/j.watres.2023.120434 37573843 10.1016/j.watres.2023.120434

[CR14] Chen Y, Zhi W, Wu J, Wang Y, Yang F, Wang G, Shen N (2025) Selective separation and concentration of volatile fatty Acids, Nitrogen, and phosphorus from Alkali-Fermented liquid using Flow-Electrode capacitive Deionization system. ACS ES&T Eng 5(11):2844–2854. 10.1021/acsestengg.5c00324

[CR15] Chu M-Y, Zhang L-S, Lou W-Y et al (2020) Preparation and characterization of oil rich in odd chain fatty acids from *Rhodococcus opacu*s PD630. J Am Oil Chem Soc 97:25–33. 10.1002/aocs.12304

[CR16] Cieciura-Włoch W, Borowski S, Otlewska A (2020) Biohydrogen production from fruit and vegetable waste, sugar beet pulp and corn silage via dark fermentation. Renew Energy 153:1226–1237. 10.1016/j.renene.2020.02.085

[CR17] Clausen CA, Coleman RD, Yang VW (2010) Fatty acid–based formulations for wood protection against mold and Sapstain. For Prod J 60:301–304. 10.13073/0015-7473-60.3.301

[CR18] Dahiya S, Lakshminarayanan S, Venkata Mohan S (2020) Steering acidogenesis towards selective propionic acid production using co-factors and evaluating environmental sustainability. Chem Eng J 379:122135. 10.1016/j.cej.2019.122135

[CR19] Data Insights Market (2025) Pentadecanoic Acid Strategic Roadmap: Analysis and Forecasts 2025–2033. https://www.datainsightsmarket.com/reports/pentadecanoic-acid-253213#. Accessed 16 Oct 2025

[CR20] de Vicente M, Gonzalez-Fernández C, Nicaud JM, Tomás-Pejó E (2025a) Turning residues into valuable compounds: organic waste conversion into odd-chain fatty acids via the carboxylate platform by Recombinant oleaginous yeast. Microb Cell Factories 24:32. 10.5281/zenodo.13928746 10.1186/s12934-025-02647-7 PMC11776196 39881394

[CR21] de Vicente M, González-Fernández C, Tomás-Pejó E (2025b) Downstream processes for yeast microbial oils extraction. Eukaryotic microorganisms as sources of bioproducts. Elsevier, pp 149–176. 10.1016/B978-0-443-30188-9.00008-1

[CR22] Devi NB, Pakshirajan K (2025) Diversifying product portfolio of Syngas fermentation in addition to ethanol production by using *Clostridium* species. Bioresour Technol 427:132401. 10.1016/j.biortech.2025.132401 40090495 10.1016/j.biortech.2025.132401

[CR23] Dishisha T, Jain M, Hatti-Kaul R (2024) High cell density sequential batch fermentation for enhanced propionic acid production from glucose and glycerol/glucose mixture using *Acidipropionibacterium Acidipropionici*. Microb Cell Fact 23:91. 10.1186/s12934-024-02366-5 38532467 10.1186/s12934-024-02366-5 PMC10964606

[CR24] Dong G, Zhao Y, Ding W et al (2024) Metabolic engineering of S*accharomyces cerevisiae* for de Novo production of odd-numbered medium-chain fatty acids. Metab Eng 82:100–109. 10.1016/j.ymben.2024.01.009 38325640 10.1016/j.ymben.2024.01.009

[CR25] Duan Y, Chen L, Ma L et al (2025) CRISPR/Cas9-mediated metabolic engineering for enhanced PUFA production in *Schizochytrium limacinum*. Chem Eng J 164320. 10.1016/j.cej.2025.164320

[CR26] El Kantar S, Koubaa M (2022) Valorization of low-cost substrates for the production of odd chain fatty acids by the oleaginous yeast *Yarrowia lipolytica*. Fermentation 8:284. 10.3390/fermentation8060284

[CR27] Fang L, Fan J, Luo S et al (2021) Genome-scale target identification in *Escherichia coli* for high-titer production of free fatty acids. Nat Commun 12:4976. 10.1038/s41467-021-25243-w 34404790 10.1038/s41467-021-25243-w PMC8371096

[CR28] Fernández-Blanco C, Veiga MC, Kennes C (2023) Effect of pH and medium composition on chain elongation with *Megasphaera hexanoica* producing C4-C8 fatty acids. Front Microbiol 14:1281103. 10.3389/fmicb.2023.1281103 38029098 10.3389/fmicb.2023.1281103 PMC10653306

[CR29] Gao R, Li Z, Zhou X et al (2020) Enhanced lipid production by *Yarrowia lipolytica* cultured with synthetic and waste-derived high-content volatile fatty acids under alkaline conditions. Biotechnol Biofuels 13:3. 10.1186/s13068-019-1645-y 31911818 10.1186/s13068-019-1645-y PMC6945533

[CR30] Gonçalves MJA, González-Fernández C, Greses S (2024) Unravelling the effects of short-term starvation and pH disturbances in stable volatile fatty acids production. J Clean Prod 476:143747. 10.1016/j.jclepro.2024.143747

[CR31] Gonçalves MJA, González-Fernández C, Greses S (2025a) Exploring anaerobic fermentation stability against a temperature perturbation: process indicators and recovery strategies. Chemosphere 387:144669. 10.1016/j.chemosphere.2025.144669 40876119 10.1016/j.chemosphere.2025.144669

[CR32] Gonçalves MJA, Greses S, Kanine O et al (2025b) Upscaling volatile fatty acids production: demonstrating the reliability of anaerobic fermentation of food wastes from the lab towards industrial implementation. Sci Tot Environ 985:179735. 10.1016/j.scitotenv.2025.179735 10.1016/j.scitotenv.2025.179735 40424900

[CR33] González-Fernández MJ, Fabrikov D, Lyashenko S, Ferrón-Carrillo F, Guil-Guerrero JL (2020) Highly concentrated very long-chain PUFA obtainment by Urea complexation methodology. Environ Technol Innov 18:100736. 10.1016/j.eti.2020.100736

[CR34] Gonzalez-Garcia R, McCubbin T, Navone L et al (2017) Microbial propionic acid production. Fermentation 3:21. 10.3390/fermentation3020021

[CR35] Gorbunov DN, Terenina MV, Kardasheva YS et al (2017) Oxo processes involving ethylene (a review). Pet Chem 57:1137–1140. 10.1134/S0965544117060159

[CR36] Greses S, Tomás-Pejó E, González-Fernández C (2022) Statistical correlation between waste macromolecular composition and anaerobic fermentation temperature for specific short-chain fatty acid production. Environ Res 206:112288. 10.1016/j.envres.2021.112288 34717941 10.1016/j.envres.2021.112288

[CR37] Greses S, Llamas M, Kaoutar A, González-Fernández C (2025) Vinasses valorization into short-chain fatty acids: Microbiome robustness against process variations. Bioresour Bioprocess 12:26. 10.1186/s40643-025-00865-w 40167882 10.1186/s40643-025-00865-w PMC11961857

[CR38] Grubišić M, Šantek B, Zorić Z et al (2022) Bioprospecting of microalgae isolated from the Adriatic sea: characterization of biomass, pigment, lipid and fatty acid composition, and antioxidant and antimicrobial activity. Molecules 27:1248. 10.3390/molecules27041248 35209036 10.3390/molecules27041248 PMC8875609

[CR39] Hao J, Wang H (2015) Volatile fatty acids productions by mesophilic and thermophilic sludge fermentation: biological responses to fermentation temperature. Bioresour Technol 175:367–373. 10.1016/j.biortech.2014.10.106 25459844 10.1016/j.biortech.2014.10.106

[CR40] Hermansen C, Siao R, Chua GG et al (2025) Short-chain fatty acid utilization in *Cyberlindnera jadinii* for single-cell protein and odd-chain fatty acid production. Microorganisms 13:1558. 10.3390/microorganisms13071558 40732067 10.3390/microorganisms13071558 PMC12299025

[CR41] Huang X, Dong W, Wang H, Feng Y (2018) Role of acid/alkali-treatment in primary sludge anaerobic fermentation: insights into microbial community structure, functional shifts and metabolic output by high-throughput sequencing. Bioresour Technol 249:943–952. 10.1016/j.biortech.2017.10.104 29145121 10.1016/j.biortech.2017.10.104

[CR42] Ingram LO, Chevalier LS, Gabba EJ et al (1977) Propionate-induced synthesis of odd-chain-length fatty acids by *Escherichia coli*. J Bacteriol 131:1023–1025. 10.1128/jb.131.3.1023-1025.1977 330493 10.1128/jb.131.3.1023-1025.1977 PMC235565

[CR43] Jänisch T, Reinhardt S, Pohsner U, Böringer S, Bolduan R, Steinbrenner J, Oechsner H (2019) Separation of volatile fatty acids from biogas plant hydrolysates. Sep Purif Technol 223:264–273. 10.1016/j.seppur.2019.04.066

[CR44] Janßen HJ, Ibrahim MHA, Bröker D, Steinbüchel A (2013) Optimization of macroelement concentrations, pH and osmolarity for triacylglycerol accumulation in *Rhodococcus opacus* strain PD630. AMB Expr 3:38. 10.1186/2191-0855-3-38 10.1186/2191-0855-3-38 PMC3723911 23855965

[CR45] Jeon BS, Choi O, Um Y, Sang B-I (2016) Production of medium-chain carboxylic acids by *Megasphaera* sp. MH with supplemental electron acceptors. Biotechnol Biofuels 9:129. 10.1186/s13068-016-0549-3 27340431 10.1186/s13068-016-0549-3 PMC4918077

[CR46] Jiménez-Páez E, Serrano A, Purswani J et al (2023) Impact on the microbial population during biological volatile fatty acid production from Olive mill solid waste. Environ Technol Innov 32:103409. 10.1016/j.eti.2023.103409

[CR47] Jin Y, Lu Y (2023) Syntrophic propionate oxidation: one of the rate-limiting steps of organic matter decomposition in anoxic environments. Appl Environ Microbiol 89. 10.1128/aem.00384-23 10.1128/aem.00384-23 PMC10231205 37097179

[CR48] Khafipour A, Jordaan EM, Flores-Orozco D et al (2020) Response of microbial community to induced failure of anaerobic digesters through overloading with propionic acid followed by process recovery. Front Bioeng Biotechnol 8:604838. 10.3389/fbioe.2020.604838 33363133 10.3389/fbioe.2020.604838 PMC7759631

[CR49] Kim H, Jeon BS, Sang B In (2019) An efficient new process for the selective production of odd-chain carboxylic acids by simple carbon elongation using *Megasphaera hexanoica*. Sci Rep 9:11999. 10.1038/s41598-019-48591-6 31427713 10.1038/s41598-019-48591-6 PMC6700076

[CR50] Kolouchová I, Schreiberová O, Sigler K et al (2015) Biotransformation of volatile fatty acids by oleaginous and non-oleaginous yeast species. FEMS Yeast Res 15. 10.1093/femsyr/fov076 10.1093/femsyr/fov076 26323601

[CR51] Krikigianni E, Matsakas L, Rova U et al (2022) Investigating the bioconversion potential of volatile fatty acids: use of oleaginous yeasts *Rhodosporidium toruloides* and *Cryptococcus curvatus* towards the sustainable production of biodiesel and Odd-Chain fatty acids. Appl Sci (Switzerland) 12. 10.3390/app12136541

[CR52] Krink-Koutsoubelis N, Loechner AC, Lechner A et al (2018) Engineered production of short-chain acyl-coenzyme A esters in *Saccharomyces cerevisiae*. ACS Synth Biol 7:1105–1115. 10.1021/acssynbio.7b00466 29498824 10.1021/acssynbio.7b00466

[CR53] Kurniawan E, O-Thong S, Cheirsilp B, Gagnon Y (2024) Optimizing continuous medium-chain fatty acid production from biohydrogenic palm oil mill effluent: operational parameters and microbial dynamics. J Clean Prod 436:140670. 10.1016/j.jclepro.2024.140670

[CR54] Lago A, Greses S, Moreno I, González-Fernández C (2025) Up-flow anaerobic sludge blanket bioreactor for the production of carboxylates: effect of inocula on process performance and microbial communities. Bioresour Bioprocess 12:6. 10.1186/s40643-025-00839-y 39853523 10.1186/s40643-025-00839-y PMC11759735

[CR55] Lazar Z, Dulermo T, Neuvéglise C et al (2014) Hexokinase—a limiting factor in lipid production from fructose in *Yarrowia lipolytica*. Metab Eng 26:89–99. 10.1016/j.ymben.2014.09.008 25307793 10.1016/j.ymben.2014.09.008

[CR56] Li J, Ban Q, Zhang L, Jha AK (2012) Syntrophic propionate degradation in anaerobic digestion: a review. Int J Agric Biol 14:843–850. 10.1016/j.envres.2024.119717

[CR57] Liu J, Yuan M, Liu J-N, Huang X-F (2017) Bioconversion of mixed volatile fatty acids into microbial lipids by *Cryptococcus curvatus* ATCC 20509. Bioresour Technol 241:645–651. 10.1016/j.biortech.2017.05.085 28609752 10.1016/j.biortech.2017.05.085

[CR58] Llamas M, Dourou M, González-Fernández C et al (2020) Screening of oleaginous yeasts for lipid production using volatile fatty acids as substrate. Biomass Bioenergy 138:105553. 10.1016/j.biombioe.2020.105553

[CR59] Llamas M, Greses S, Tomás-Pejó E, González-Fernández C (2022) Carboxylic acids production via anaerobic fermentation: microbial communities’ responses to stepwise and direct hydraulic retention time decrease. Bioresour Technol 344:126282. 10.1016/j.biortech.2021.126282 34752887 10.1016/j.biortech.2021.126282

[CR60] Luo J, Li Y, Li Y et al (2021) Waste-to-energy: cellulase induced waste activated sludge and paper waste co-fermentation for efficient volatile fatty acids production and underlying mechanisms. Bioresour Technol 341:125771. 10.1016/j.biortech.2021.125771 34411945 10.1016/j.biortech.2021.125771

[CR61] Lv N, Cai G, Pan X et al (2022) pH and hydraulic retention time regulation for anaerobic fermentation: focus on volatile fatty acids production/distribution, microbial community succession and interactive correlation. Bioresour Technol 347:126310. 10.1016/j.biortech.2021.126310 34767905 10.1016/j.biortech.2021.126310

[CR62] Ma H, Liu H, Zhang L et al (2017) Novel insight into the relationship between organic substrate composition and volatile fatty acids distribution in acidogenic co-fermentation. Biotechnol Biofuels. 10.1186/s13068-017-0821-1 28559928 10.1186/s13068-017-0821-1 PMC5446719

[CR63] Ma W, Li J, Yang W-Q et al (2023) Efficient biosynthesis of odd-chain fatty acids via regulating the supply and consumption of propionyl-CoA in *Schizochytrium* sp. J Agric Food Chem 71:9847–9855. 10.1021/acs.jafc.3c03156 37326390 10.1021/acs.jafc.3c03156

[CR64] Magdalena JA, Greses S, González-Fernández C (2019) Impact of organic loading rate in volatile fatty acids production and population dynamics using microalgae biomass as substrate. Sci Rep 9:18374. 10.1038/s41598-019-54914-4 31804573 10.1038/s41598-019-54914-4 PMC6895168

[CR65] Magdalena JA, Greses S, González-Fernández C (2020) Anaerobic degradation of protein-rich biomass in an UASB reactor: organic loading rate effect on product output and microbial communities dynamics. J Environ Manage 274:111201. 10.1016/j.jenvman.2020.111201 32798846 10.1016/j.jenvman.2020.111201

[CR66] Meng Q, Ding W, Cui H et al (2025) Reprogramming yeast metabolism to alter fatty acid profiles from even-chain to odd-chain configuration. Bioresour Technol 132858. 10.1016/j.biortech.2025.132858 40545048 10.1016/j.biortech.2025.132858

[CR67] Messina E, de Souza CP, Cappella C et al (2023) Genetic inactivation of the carnitine/acetyl-carnitine mitochondrial carrier of *Yarrowia lipolytica* leads to enhanced odd-chain fatty acid production. Microb Cell Fact 22:128. 10.1186/s12934-023-02137-8 37443049 10.1186/s12934-023-02137-8 PMC10339547

[CR68] Micalizzi EW, Golshani A, Smith ML (2021) Propionic acid disrupts endocytosis, cell cycle, and cellular respiration in yeast. BMC Res Notes 14:335. 10.1186/s13104-021-05752-z 34454571 10.1186/s13104-021-05752-z PMC8403364

[CR69] Oh HW, Lee SC, Woo HC, Kim YH (2022) Energy-efficient recovery of fermented butyric acid using octyl acetate extraction. Biotechnology for Biofuels and Bioproducts 15(1):46. 10.1186/s13068-022-02146-6 35524283 10.1186/s13068-022-02146-6 PMC9074251

[CR70] Oliver L, Fernández-de-Castro L, Dietrich T et al (2022) Production of docosahexaenoic acid and odd-chain fatty acids by microalgae *Schizochytrium limacinu*m grown on waste-derived volatile fatty acids. Appl Sci 12:3976. 10.3390/app12083976

[CR71] Park Y, Nicaud J (2020a) Screening a genomic library for genes involved in propionate tolerance in *Yarrowia lipolytica*. Yeast 37:131–140. 10.1002/yea.3431 31293017 10.1002/yea.3431

[CR72] Park Y-K, Nicaud J-M (2020b) Metabolic engineering for unusual lipid production in *Yarrowia lipolytica*. Microorganisms 8:1937. 10.3390/microorganisms8121937 33291339 10.3390/microorganisms8121937 PMC7762315

[CR73] Park Y-K, Dulermo T, Ledesma-Amaro R, Nicaud J-M (2018) Optimization of odd chain fatty acid production by *Yarrowia lipolytica*. Biotechnol Biofuels 11:158. 10.1186/s13068-018-1154-4 29930704 10.1186/s13068-018-1154-4 PMC5991449

[CR74] Park Y, Ledesma-Amaro R, Nicaud J-M (2020) *De novo* biosynthesis of odd-chain fatty acids in *Yarrowia lipolytica* enabled by modular pathway engineering. Front Bioeng Biotechnol 7:484. 10.3389/fbioe.2019.00484 32039184 10.3389/fbioe.2019.00484 PMC6987463

[CR75] Park Y-K, Bordes F, Letisse F, Nicaud J-M (2021) Engineering precursor pools for increasing production of odd-chain fatty acids in *Yarrowia lipolytica*. Metab Eng Commun 12:e00158. 10.1016/j.mec.2020.e00158 33391990 10.1016/j.mec.2020.e00158 PMC7773535

[CR76] Paul SK, Gupta DR, Ino M et al (2025) 3-methyl pentanoic acid suppress Gray mold disease potentially targeting cell-wall integrity (CWI) and mitogen-activated protein kinase (MAPK) pathways in *Botrytis cinerea*. BMC Microbiol 25:470. 10.1186/s12866-025-04180-4 40750846 10.1186/s12866-025-04180-4 PMC12315443

[CR77] Pervez MN, Mahboubi A, Uwineza C, Zarra T, Belgiorno V, Naddeo V, Taherzadeh MJ (2022) Factors influencing pressure-driven membrane-assisted volatile fatty acids recovery and purification-a review. Sci Total Environ 817:152993. 10.1016/j.scitotenv.2022.152993 35026250 10.1016/j.scitotenv.2022.152993

[CR78] Polat E, Genç AN, Güngör FŞ, Altınbaş M (2025) Recovery of volatile fatty acids from anaerobic fermentation broth of baker’s yeast industry effluent by liquid − liquid extraction. J Ind Eng Chem 141:431–440. 10.1016/j.jiec.2024.07.005

[CR79] Qi N, Ding W, Dong G et al (2023) *De novo* bio-production of odd‐chain fatty acids in *Saccharomyces cerevisiae* through a synthetic pathway via 3‐hydroxypropionic acid. Biotechnol Bioeng 120:852–858. 10.1002/bit.28297 36464776 10.1002/bit.28297

[CR80] Qin N, Li L, Wang Z, Shi S (2023) Microbial production of odd-chain fatty acids. Biotechnol Bioeng 120:917–931. 10.1002/bit.28308 36522132 10.1002/bit.28308

[CR81] Reddy MV, Nandan Reddy VU, Chang Y-C (2022) Integration of anaerobic digestion and chain elongation technologies for biogas and carboxylic acids production from cheese whey. J Clean Prod 364:132670. 10.1016/j.jclepro.2022.132670

[CR82] Regueira A, Bevilacqua R, Lema JM et al (2020) A metabolic model for targeted volatile fatty acids production by cofermentation of carbohydrates and proteins. Bioresour Technol 298:122535. 10.1016/j.biortech.2019.122535 31865254 10.1016/j.biortech.2019.122535

[CR83] Runguphan W, Keasling JD (2014) Metabolic engineering of *Saccharomyces cerevisiae* for production of fatty acid-derived biofuels and chemicals. Metab Eng 21:103–113. 10.1016/j.ymben.2013.07.003 23899824 10.1016/j.ymben.2013.07.003

[CR84] Schweizer E, Hofmann J (2004) Microbial type I fatty acid synthases (FAS): major players in a network of cellular FAS systems. Microbiol Mol Biol Rev 68:501–517. 10.1128/MMBR.68.3.501-517.2004 15353567 10.1128/MMBR.68.3.501-517.2004 PMC515254

[CR85] Seeliger S, Janssen PH, Schink B (2002) Energetics and kinetics of lactate fermentation to acetate and propionate via methylmalonyl-CoA or acrylyl-CoA. FEMS Microbiol Lett 211:65–70. 10.1111/j.1574-6968.2002.tb11204.x 12052552 10.1111/j.1574-6968.2002.tb11204.x

[CR86] Sun J, Zhang L, Loh K-C (2021) Review and perspectives of enhanced volatile fatty acids production from acidogenic fermentation of lignocellulosic biomass wastes. Bioresour Bioprocess. 10.1186/s40643-021-00420-3 38650255 10.1186/s40643-021-00420-3 PMC10992391

[CR87] Sun H, Zhang X, Cui M, Liu G, Liu H, Huang S, Liu H (2023) Separation of nutrients and acetate from sewage sludge fermentation liquid in flow-electrode capacitive Deionization system: competitive mechanisms of ions and influence of activated carbon. Bioresour Technol 390:129864. 10.1016/j.biortech.2023.129864 37839646 10.1016/j.biortech.2023.129864

[CR88] Tabaa Chalabi N, El Kantar S, De Pires C et al (2024) Improving the synthesis of odd-chain fatty acids in the oleaginous yeast *Yarrowia lipolytica*. Fermentation 10:597. 10.3390/fermentation10120597

[CR89] Tomás-Pejó E, Morales-Palomo S, González-Fernández C (2021) Microbial lipids from organic wastes: outlook and challenges. Bioresour Technol 323:124612. 10.1016/j.biortech.2020.124612 33418352 10.1016/j.biortech.2020.124612

[CR90] Tomás-Pejó E, González-Fernández C, Greses S et al (2023) Production of short-chain fatty acids (SCFAs) as chemicals or substrates for microbes to obtain biochemicals. Biotechnol Biofuels 16:96. 10.1186/s13068-023-02349-5 10.1186/s13068-023-02349-5 PMC10239154 37270640

[CR91] Tseng H-C, Prather KLJ (2012) Controlled biosynthesis of odd-chain fuels and chemicals via engineered modular metabolic pathways. Proc Natl Acad Sci 109:17925–17930. 10.1073/pnas.1209002109 23071297 10.1073/pnas.1209002109 PMC3497732

[CR92] Verified Market Reports (2025) Heptadecanoic Acid Market Size, Competitive Overview, Trends & Forecast 2033. https://www.verifiedmarketreports.com/product/heptadecanoic-acid-market/. Accessed 16 Oct 2025

[CR93] Voss I, Steinbüchel A (2001) High cell density cultivation of *Rhodococcus opacus* for lipid production at a pilot-plant scale. Appl Microbiol Biotechnol 55:547–555. 10.1007/s002530000576 11414319 10.1007/s002530000576

[CR94] Wang F, Bi Y, Diao J et al (2019) Metabolic engineering to enhance biosynthesis of both docosahexaenoic acid and odd-chain fatty acids in *Schizochytrium* sp. S31. Biotechnol Biofuels 12:141. 10.1186/s13068-019-1484-x 31182976 10.1186/s13068-019-1484-x PMC6555965

[CR95] Weimer PJ, Nerdahl M, Brandl DJ (2015) Production of medium-chain volatile fatty acids by mixed ruminal microorganisms is enhanced by ethanol in co-culture with *Clostridium kluyveri*. Bioresour Technol 175:97–101. 10.1016/j.biortech.2014.10.054 25459809 10.1016/j.biortech.2014.10.054

[CR96] Westerholm M, Calusinska M, Dolfing J (2022) Syntrophic propionate-oxidizing bacteria in methanogenic systems. FEMS Microbiol Rev 46:1–26. 10.1093/femsre/fuab057 10.1093/femsre/fuab057 PMC8892533 34875063

[CR97] Wu H, San K-Y (2014a) Efficient odd straight medium chain free fatty acid production by metabolically engineered *Escherichia coli*. Biotechnol Bioeng 111:2209–2219. 10.1002/bit.25296 24889416 10.1002/bit.25296

[CR98] Wu H, San K-Y (2014b) Engineering *Escherichia coli* for odd straight medium chain free fatty acid production. Appl Microbiol Biotechnol 98:8145–8154. 10.1007/s00253-014-5882-5 25030454 10.1007/s00253-014-5882-5

[CR99] Xu R, Fanf S, Zhang L et al (2021) Distribution patterns of functional microbial community in anaerobic digesters under different operational circumstances: A review. Bioresour Technol 341:125823. 10.1016/j.biortech.2021.125823 34454239 10.1016/j.biortech.2021.125823

[CR100] Xue J, Balamurugan S, Li T et al (2021) Biotechnological approaches to enhance biofuel producing potential of microalgae. Fuel 302:121169. 10.1016/j.fuel.2021.121169

[CR101] Zeng X, Danquah MK, Chen XD, Lu Y (2011) Microalgae bioengineering: from CO_2_ fixation to biofuel production. Renew Sustain Energy Rev 15:3252–3260. 10.1016/j.rser.2011.04.014

[CR102] Zhang L-S, Xu P, Chu M-Y et al (2019) Using 1-propanol to significantly enhance the production of valuable odd-chain fatty acids by *Rhodococcus opacus* PD630. World J Microbiol Biotechnol 35:164. 10.1007/s11274-019-2748-0 31637528 10.1007/s11274-019-2748-0

[CR103] Zhang L-S, Liang S, Zong M-H et al (2020) Microbial synthesis of functional odd-chain fatty acids: a review. World J Microbiol Biotechnol 36:35. 10.1007/s11274-020-02814-5 32088779 10.1007/s11274-020-02814-5

[CR104] Zhang Y, Li C, Yuan Z et al (2023) Syntrophy mechanism, microbial population, and process optimization for volatile fatty acids metabolism in anaerobic digestion. Chem Eng J 452:139137. 10.1016/j.cej.2022.139137

[CR105] Ziemiński K, Frąc M (2012) Methane fermentation process as anaerobic digestion of biomass: transformations, stages and microorganisms. Afr J Biotechnol 11:4127–4139. 10.5897/AJBX11.054

